# Books and Pamphlets Received

**Published:** 1884-11-20

**Authors:** 


### BOOKS AND PAMPHLETS RECEIVED.

*The Elements of Pathology.* By Edward Rindfleisch, M. D., Professor of Pathologal Anatomy in the University of Wursburg. Translated from the German by W. H. Mercur, M. D. (University of Penn). Revised by James Tyson, M. D., Professor of General Pathology and Morbid Anatomy in the University of Pennsylvania ; one of the Physicians to the Philadelphia Hospital, etc. Cloth, pp. 263. Philadelphia : P. Blakiston, Son & Co. ; S. P. Richards & Son, Atlanta, Ga.

The few works in this country on the department of Pathology makes the present book a welcome visitor ; and the high reputation of Professor Rindfleisch will at once commend the work to the profession in this country.

*Handbook of the Diagnosis and Treatment of Skin Diseases.* By Arthur Van Harlingen, M. D , Professor of Diseases of the Skin in the Philadelphia Polyclinic and College for Graduates in Medicine; Consulting Physician to the Dispensary for Skin Diseases, Philadelphia; Physician to the Howard Hospital, etc. With two Colored Plates. Philadelphia: P. Blakiston, Son & Co., 1012 Walnut street; S. P. Richards & Son, Atlanta, Ga.

A work of 282 pages, neatly gotten out in cloth. The author states that it is written for the Practitioner, and is plain, full and satisfactory, especially on the class of skin affections most frequently met with. It is eminently practical and fully up with the latest advances in this department.

*Proceedings of the First Annual Session of the Southern Immigration Association of America, held at Nashville, Tenn., March* 11, 12, 13, 1884. *A. J. McWhorter, President, Nashville, Tenn. S. H. Nowlin, Secretary, Little Rock, Ark.*

We are indebted to the kindness of the Hon. R. B. Reppard, of Savannah, one of the Vice-Presidents of the Association, for a copy of this interesting work, containing 350 octavo pages and replete with interesting information touching the soil, climate, production, etc., of the Southern States of the Union.

The object of the Association is to secure the co-operation of the Southern States on means for developing their resources—"To establish immigrant homes at such Southern sea-ports as the managers may deem necessary; to encourage immigration to said States; to gather, publish and distribute statistics, maps and other literature upon the agricultural, mining, manufacturing, educational and other interests of the South; to make contracts for the settlement of immigrants in said States, and to establish agencies at such places, and with such powers and duties as the Association may from time to time designate."

*Opera Minora*, a Collection of Essays, Articles, Lectures and Addresses from 1866 to 1882, inclusive, by Edward C. Seguin, Chemical Professor of Diseases of the Mind and Nervous System in the College of Physicians and Surgeons, New York, etc. C. Putnam & Sons, New York.

This work contains 683 large octavo pages, and is a work of marked ability and interest. Dr. Seguin, the author, is a gentleman of learning and of national reputation, particularly in the line of nervous affections. His papers at the American Medical Association and other contributions to the Medical Press have secured for him an enviable position among the distinguished members of the profession. The present book is a compilation of his various medical contributions and will doubtless be gladly welcomed by progressive men in the profession throughout the world.

*Quarantine and Commerce.* Their antagonism destructive to the prosperity of city and state. A reconciliation an imperative necessity. How this may be accomplished. Remarks of the President of the Board of Health of the State of Louisiana before the representatives of the exchanges and other commercial bodies. Arranged chiefly from the reports of proceedings of a called meeting of the New Orleans Produce Exchange, held June 20th, 1884, as presented in the Picayune and Times-Democrat. Together with his argument before the Senate Finance Committee of the General Assembly of Louisiana, June 26th, 1884.

*Genital Reflexes*, the Result of an Abnormal Physical Condition of the Genital Organs known as Phimosis, by T. Griswold Comstock, M.A., M.D., St. Louis., Mo.

*National University*, Washington, D. C. Law Department. Announcements for 1884—1885. Law Building, 1006 E. Street, N. W. F. L. Lavender, Esq., Treasurer; Office, 480½ Louisiana Ave.

